# Pretreatment risk management of a novel nomogram model for prediction of thoracoabdominal extrahepatic metastasis in primary hepatic carcinoma

**DOI:** 10.1186/s12967-019-1861-z

**Published:** 2019-04-08

**Authors:** Jia Hu, Ting Wang, Kun-He Zhang, Yi-Ping Jiang, Song Xu, Si-Hai Chen, Yu-Ting He, Hai-Liang Yuan, Yu-Qi Wang

**Affiliations:** 1 Department of Gastroenterology, The First Affiliated Hospital of Nanchang University, 17 Yongwai Zheng Street, Nanchang, 330006 China; 2 Department of Gastroenterology, Jiangxi Institute of Gastroenterology and Hepatology, 17 Yongwai Zheng Street, Nanchang, 330006 China; 3 Department of Gastroenterology, The Affiliated Hospital of Jiangxi University of Traditional Chinese Medicine, 445 Bayi Road, Nanchang, 330006 China

**Keywords:** Primary hepatic carcinoma, Thoracoabdominal extrahepatic metastasis, Nomogram, Pretreatment risk management, Individualized clinical decision-making

## Abstract

**Background:**

Extrahepatic metastasis is the independent risk factor of poor survival of primary hepatic carcinoma (PHC), and most occurs in the chest and abdomen. Currently, there is still no available method to predict thoracoabdominal extrahepatic metastasis in PHC. In this study, a novel nomogram model was developed and validated for prediction of thoracoabdominal extrahepatic metastasis in PHC, thereby conducted individualized risk management for pretreatment different risk population.

**Methods:**

The nomogram model was developed in a primary study that consisted of 330 consecutive pretreatment patients with PHC. Large-scale datasets were extracted from clinical practice. The nomogram was based on the predictors optimized by data dimension reduction through Lasso regression. The prediction performance was measured by the area under the receiver operating characteristic (AUROC), and calibrated to decrease the overfit bias. Individualized risk management was conducted by weighing the net benefit of different risk population via decision curve analysis. The prediction performance was internally and independently validated, respectively. An independent-validation study using a separate set of 107 consecutive patients.

**Results:**

Four predictors from 55 high-dimensional clinical datasets, including size, portal vein tumor thrombus, infection, and carbohydrate antigen 125, were incorporated to develop a nomogram model. The nomogram demonstrated valuable prediction performance with AUROC of 0.830 (0.803 in internal-validation, and 0.773 in independent-validation, respectively), and fine calibration. Individual risk probability was visually scored. Weighing the net benefit, threshold probability was classified for three-independent risk population, which was < 19.9%, 19.9–71.8% and > 71.8%, respectively. According to this classification, pretreatment risk management was based on a treatment-flowchart for individualized clinical decision-making.

**Conclusions:**

The proposed nomogram is a useful tool for pretreatment risk management of thoracoabdominal extrahepatic metastasis in PHC for the first time, and may handily facilitate timely individualized clinical decision-making for different risk population.

**Electronic supplementary material:**

The online version of this article (10.1186/s12967-019-1861-z) contains supplementary material, which is available to authorized users.

## Background

Primary hepatic carcinoma (PHC), including hepatocellular carcinoma (HCC) and intrahepatic cholangiocarcinoma (ICC), is one of the commonest cancers leading to death worldwide. The clinic strategy and prognosis of patients with PHC strongly depends on the tumor stage at the time of diagnosis [1]. Patients with advanced-stage PHC, defined according to the presence of extrahepatic metastasis, are generally considered candidates for palliative therapy instead of curative treatment, with poor prognosis (median survival of < 1 year) [2] in contrast to the > 70% 5-year survival of early-stage PHC without extrahepatic metastasis [3]. Extrahepatic metastasis is the independent predictor of poor survival of PHC [4], and the most frequent metastatic sites occur in chest and abdomen (exceeding 90% of extrahepatic metastasis), [5, 6] including lungs, lymph nodes, bones (ribs and thoracolumbar vertebraes), adrenal glands, gastrointestinal tract, and pleuroperitoneum. Therefore, warning for extrahepatic spread in the chest and abdomen is crucial to determine the optimal clinical strategy for improving the prognosis of pretreatment patients with PHC.

The current diagnosis of extrahepatic metastasis of PHC mainly relies on biomarkers, biopsy, and imaging scanning. Several biomarkers had been proposed as predictors, such as alpha-fetoprotein (AFP) mRNA, glypican-3, CK19, CD44, and vascular endothelial growth factor [7–9]. However, their pragmatic value remains controversial so that they still not serve for the clinical applications. The biopsy may result in additional injury to the patient and be not suitable for repeated. At present, the most valuable diagnostic strategy are comprehensive scanning of medical imaging and regular monitoring. However, these workups are costly, complicated, time-consuming, and probably unnecessary for majority which may not benefit more than tumor treatments after essential examination. There is still no available method to predict thoracoabdominal extrahepatic metastasis in PHC. Therefore, it is necessary to conduct risk prediction for thoracoabdominal extrahepatic metastasis in PHC, in order to facilitate the individualized clinical risk management for different pretreatment population.

In recent years, some new techniques provide a powerful tool for multivariate combination analyses and strongly facilitate the progress of diagnostics. A typical example is the combination of logistic regression analysis with the least absolute shrinkage and selection operator (LASSO) [10, 11] for data dimension reduction and variable selection, nomogram [12, 13] for visionally scoring probability, and decision curve analysis (DCA) [14] for the net benefit of clinical decision-making, which has provided a systematic strategy for multivariate combination analyses and timely the individualized decision-making. Huang et al. [15] utilized the strategy to develop a nomogram capable of predicting lymph node metastasis in patients with colorectal cancer, and assessing the net benefit at different threshold probabilities.

In the present study, we aimed to develop and validate a novel nomogram for risk management of thoracoabdominal extrahepatic metastasis in PHC for the first time. We also attempted to facilitate timely individualized clinical decision-making based on pretreatment risk management for different risk population.

## Methods

### Patients

**Fig. 1:**
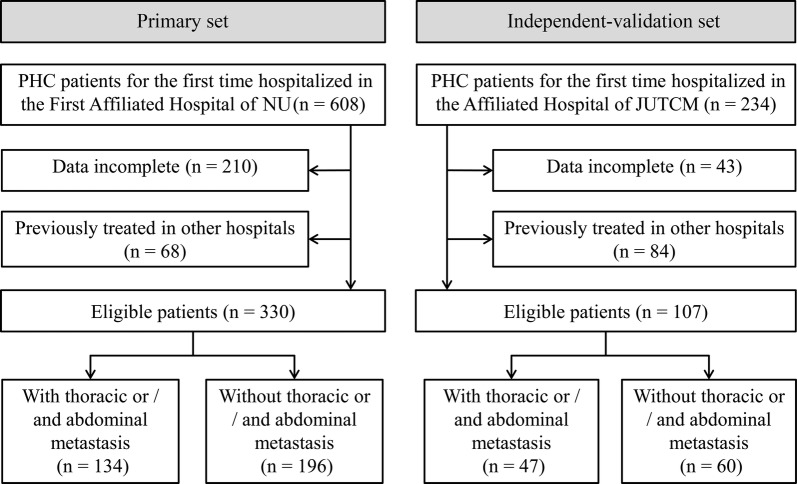
Flowchart of inclusion and exclusion of patients with primary hepatic carcinoma (PHC). NU, Nanchang University; JUTCM, Jiangxi University of Traditional Chinese Medicine

Consecutive patients with PHC for the first time hospitalized in the First Affiliated Hospital of Nanchang University from October 2015 to April 2018 were retrospectively enrolled as the primary study. PHC was diagnosed with pathology and/or dynamic contrast cross-sectional imaging such as computed tomography (CT) and magnetic resonance imaging (MRI) [16, 17]. Thoracoabdominal extrahepatic metastasis was diagnosed via pathology and/or imaging examinations (X-ray, CT, MRI, and/or bone scan). Patients were excluded from this study if they: (1) had incomplete data; (2) had undergone tumor treatments before this hospitalization. An independent-validation study of consecutive patients with PHC in the Affiliated Hospital of Jiangxi University of Traditional Chinese Medicine was enrolled from January 2016 to August 2018 using the same criteria as that for the primary study. The inclusion–exclusion process of patients is summarized in Fig. 1.

Collectable large-scale datasets were extracted from clinical practice, including demographics, etiology, intrahepatic tumor lesions, clinical conditions, tumor markers, blood cell analyses, blood biochemistry (hepatic function, renal function, electrolytes, lipid, and glucose), and coagulation function. Infections were diagnosed according to the criteria of the Center for Diseases Control, [18] including spontaneous bacterial peritonitis (SBP), systemic inflammatory response syndrome (SIRS), and sepsis, but excluding hepatotropic virus infection. SBP was diagnosed if the ascetic fluid polymorph nuclear cell count was > 250/mm^3^ in the absence of an intra-abdominal source of infection, regardless of positive culture [19]. SIRS and sepsis were diagnosed based on the definition of the American College of Chest Physicians/Society of Critical Care Medicine and the diagnostic criteria in the committee of the Consensus Conference [20]. The liver disease will be considered to be related to alcohol, regardless of positive other risk factors, if the mean pure alcohol consumption in individuals aged > 5 years, and > 40 g/day in male (or > 20 g/day in female) [21].

### Statistical analysis

Statistical analyses were performed using SPSS 24.0 (IBM, USA) and R software (version 3.5.1; http://www.Rproject.org). Continuous variables were expressed as mean and standard deviation (mean ± SD) and compared between two groups using independent-sample *t* test. Categorical variables were expressed as frequency and/or percentage and compared by using the Pearson’s Chi squared test. *P* < 0.05 was considered significant.

### Development and risk management of a nomogram model

Based on the primary data set, the LASSO [10, 11] method was used to data dimension reduction and screen the optimal predictors for modeling. Patients in the primary study were randomly divided into training and internal-validation sets by a ratio of 7:3. Through the binary multivariate logistic regression analysis, a model was developed in the training set. Internal and independent validations were performed using the internal and independent-validation data sets, respectively. The logistic regression formula formed in the training set was applied to all patients of two validation sets, with risk probability for each patient calculated.

To quantify the prediction performance, the receiver operating characteristics (ROC) curve and the area under the curve (AUROC) were used to evaluate the diagnostic value of the model for discriminating the metastasis from non-metastasis to determine the cut-off value for calculating sensitivity, specificity, accuracy, and positive/negative predictive values. Calibration curves were plotted to assess the calibration of each set, accompanied with the Hosmer–Lemeshow test (*P *> 0.05 implies that the model calibrates perfectly) [22]. A nomogram was presented for visually scoring individual risk probability of the thoracoabdominal extrahepatic metastasis [12, 13]. DCA was conducted to quantify the net benefits at different risk threshold probabilities [14]. According to weighing the net benefits, pretreatment risk management was based on a treatment-flow chart for individualized clinical decision-making.

## Results

### Demographic and clinical characteristics of the patients

A total of 330 eligible patients from 608 consecutive patients were enrolled in the primary study and 107 from 234 consecutive into the independent-validation study (Fig. 1). Metastatic positivity was 40.6% and 43.9% in primary and independent-validation sets, respectively, with no significant differences between the two sets in extrahepatic metastasis of chest and abdomen (*P* = 0.545). Demographic and clinical characteristics of the patients in primary and independent-validation sets are given in Table 1 (Additional file 1: Tables S1 and S2).

**Table 1:** Characteristics of patients

	Primary set			Independent-validation set		
	Metastasis (n = 134)	Non-metastasis (n = 196)	*P*	Metastasis (n = 47)	Non-metastasis (n = 60)	*P*
Age (mean ± SD, years)	56.8 ± 11.9	55.4 ± 11.8	0.302	60.6 ± 14.0	64.0 ± 12.3	0.182
Gender [male/female, n (%)]	97 (72.4)/37 (27.6)	165 (84.2)/31 (15.8)	0.009	37 (78.7)/10 (21.3)	47 (78.3)/13 (21.7)	0.961
Metastatic site [n (%)]						
Lymph node	78 (58.2)	–	–	18 (38.3)	–	–
Lung	14 (10.5)	–	–	7 (14.9)	–	–
Gastrointestinal tract	7 (5.2)	–	–	1 (2.1)	–	–
Adrenal gland	5 (3.7)	–	–	5 (10.6)	–	–
Bone	4 (3.0)	–	–	3 (6.4)	–	–
Pleuroperitonea	2 (1.5)	–	–	2 (4.3)	–	–
Multiple sites	24 (17.9)	–	–	11 (23.4)	–	–
Size (mean ± SD, cm)	7.6 ± 3.9	5.5 ± 3.6	< 0.001	7.7 ± 3.6	5.9 ± 4.0	0.018
PVTT [n (%)]	86 (64.2)	60 (30.6)	< 0.001	16 (34.0)	8 (13.3)	0.011
Infection	35 (26.1)	10 (5.1)	< 0.001	23 (48.9)	14 (23.3)	0.006
CA125 (U/mL)						
Levels (mean ± SD)	138.1 ± 249.9	85.5 ± 218.9	0.049	292.3 ± 349.5	170.3 ± 268.7	0.051
Positive rates [> 13.9*, n (%)]	111 (82.9)	100 (51.0)	< 0.001	24 (51.1)	43 (71.7)	0.029
AFP (ng/mL)						
Levels (mean ± SD)	476.6 ± 680.1	384.9 ± 513.3	0.187	511.4 ± 735.9	422.6 ± 651.2	0.510
Positive rates [n (%)]						
≥ 20	70 (52.2)	108 (55.1)	0.608	32 (68.1)	35 (58.3)	0.301
≥ 200	57 (42.5)	71 (36.2)	0.248	26 (55.3)	35 (58.3)	0.755
≥ 400	84 (62.7)	132 (67.3)	0.382	32 (68.1)	40 (66.7)	0.877

*SD* standard deviation, *PVTT* portal vein tumor thrombus, *CA125* carbohydrate antigen 125, *AFP* alpha-fetoprotein

*P* value is derived from the univariate association analyses between metastasis group and non-metastasis group; Size: the maximum diameter of intrahepatic lesions; *: best cut-off value according to receiver operating characteristic (ROC) curve in primary set

In order to more effectively manage the data of tumor markers, we attempted to convert continuous variables into categorical variables on the basis of different threshold stratifications while retaining original serum concentrations. AFP was stratified by 20, 200 and 400 ng/mL, respectively. Carbohydrate antigen (CA) 125 was stratified by 13.9 U/mL, which was its best cut-off value determined by ROC curve in primary set. The concentrations and positive rates of serum tumor markers in metastasis and non-metastasis groups are shown in Table 1 (Additional file 1: Table S3).

### Predictor selection and model development

**Fig. 2:**
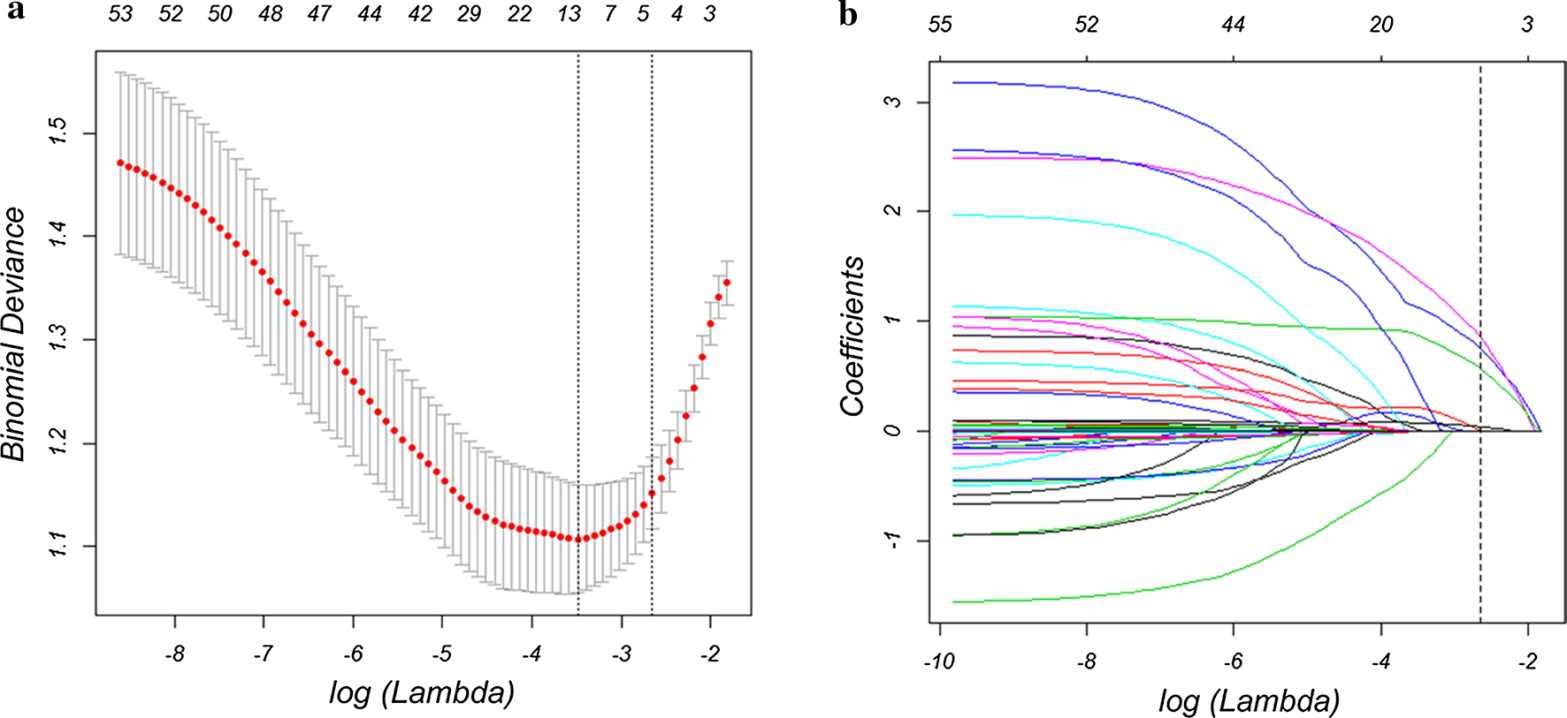
Predictor selection by the least absolute shrinkage and selection operator (LASSO). **a** Parameter (Lambda) selection by LASSO adopted tenfold cross-validation via minimum criteria. Dotted vertical lines were drawn at the optimal values by adopting the minimum criteria and the 1 standard error of the minimum criteria (the 1 − SE criteria). The Lambda value of 0.071, with log (Lambda), − 1.478 was chosen (1 − SE criteria) by tenfold cross-validation. **b** LASSO coefficient profile plot of 55 variables against the log (Lambda) sequence. Vertical line was drawn at optimal Lambda value with 4 nonzero coefficients by tenfold cross-validation

A total of 55 high-dimensional clinical data were incorporated in the LASSO regression and four best predictors were selected: size, portal vein tumor thrombus (PVTT), infection, and CA125 (Fig. 2).

Patients in the primary study were randomly divided into training (225 cases, approximately 70%) and internal-validation sets (105 cases, the others). The four predictors (size, PVTT, infection, and CA125) were incorporated to develop a predictive model for thoracoabdominal metastasis in PHC via binary logistic regression based on the training set. The detailed parameters of predictors in the model are shown in Table 2.

**Table 2:** Detailed parameters of the predictors in the model

	β	Odds ratio (95% CI)	*P*
Intercept	− 3.013		< 0.001
Size	0.122	1.129 (1.036–1.231)	0.006
PVTT	1.915	6.785 (3.463–13.293)	< 0.001
Infection	2.011	7.473 (2.685–20.804)	< 0.001
CA125	1.038	2.824 (1.403–5.686)	0.004

β is the regression coefficient. Size: the maximum diameter of intrahepatic lesions. *CI* confidence interval, *PVTT* portal vein tumor thrombus, *CA125* carbohydrate antigen 125

### Performances of prediction and calibration

**Fig. 3:**
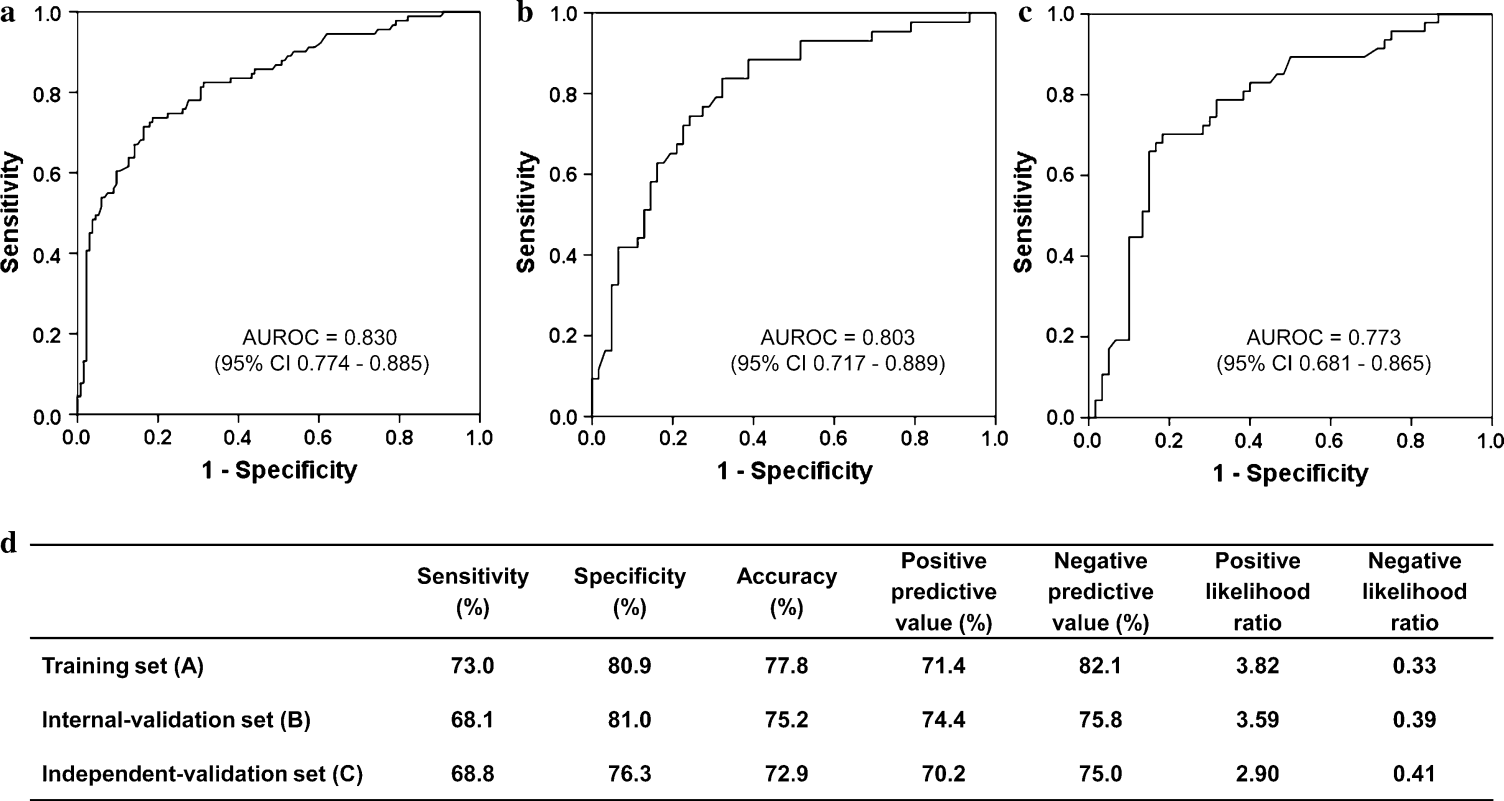
Prediction performance of the model. **a** Receiver operating characteristic (ROC) curve plot in the training set; **b** ROC curve plot in the internal-validation set; **c** ROC curve plot in the independent-validation set; **d** predictive parameters in each set of the model; *AUROC* the area under the receiver operating characteristic, *CI* confidence interval

**Fig. 4:**
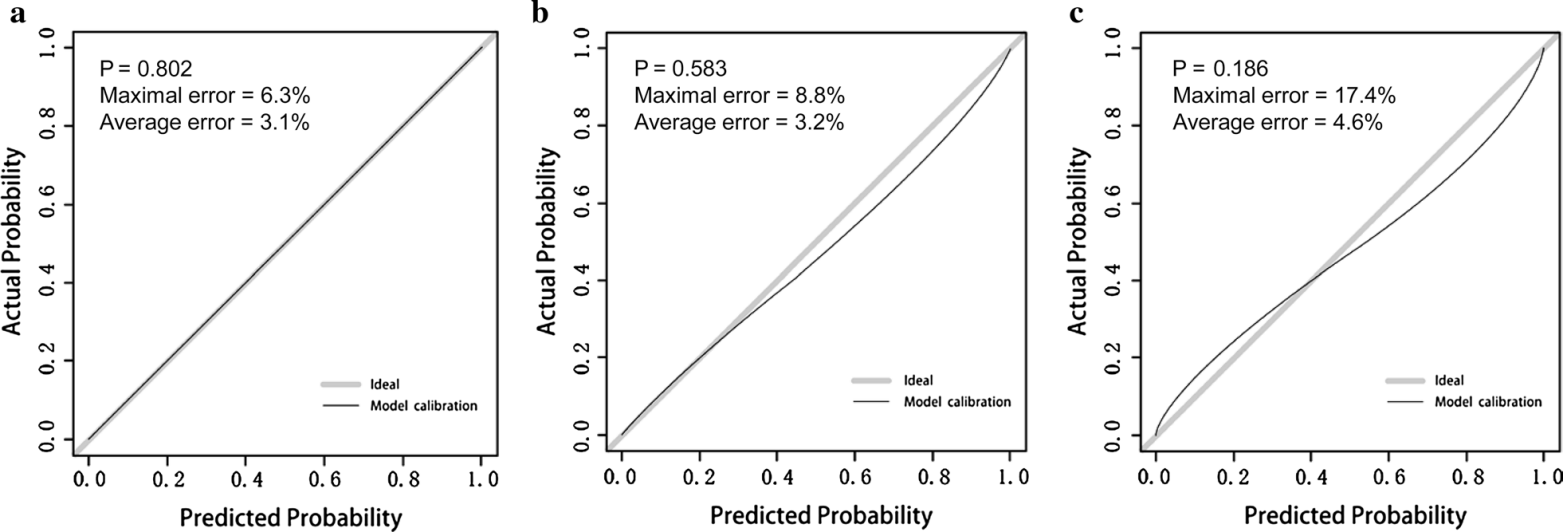
Calibration curve plot in each set. **a** the training set; **b** the internal-validation set; **c** the independent–validation set

The ROC curves and prediction performance of the model are shown in Fig. 3. The model demonstrated valuable prediction performance with AUROC of 0.830 (0.803 in internal validation, and 0.773 in independent validation, respectively). The calibration curves of the model showed good agreement between prediction and observation (Fig. 4). Good calibration was observed and the Hosmer–Lemeshow test demonstrated a nonsignificant statistic in each set (*P* > 0.05), which suggested that no departure was perfectly fit in the model.

### The presentation of a nomogram and clinical risk management

**Fig. 5:**
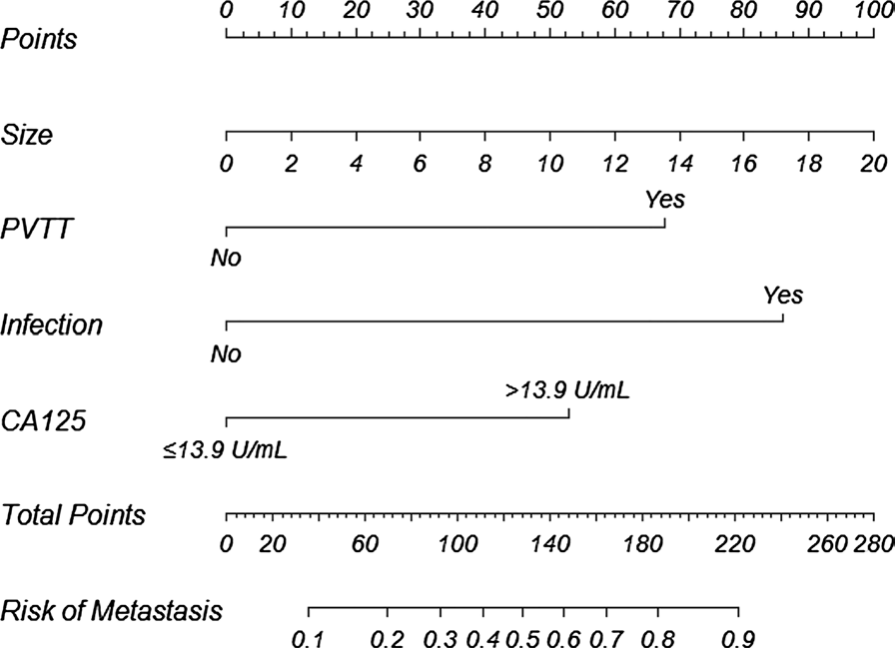
The nomogram model for quantifying individual risk of thoracoabdominal extrahepatic metastasis in PHC. For a pretreatment patient with PHC, the risk of thoracoabdominal extrahepatic metastasis according to the nomogram is the probability in “Risk of Metastasis” corresponding to “Total Points” of all four indicator points summing

Based on the predictive model, a nomogram was presented, and individual metastatic risk probabilities can be visually scored (Fig. 5).

**Fig. 6:**
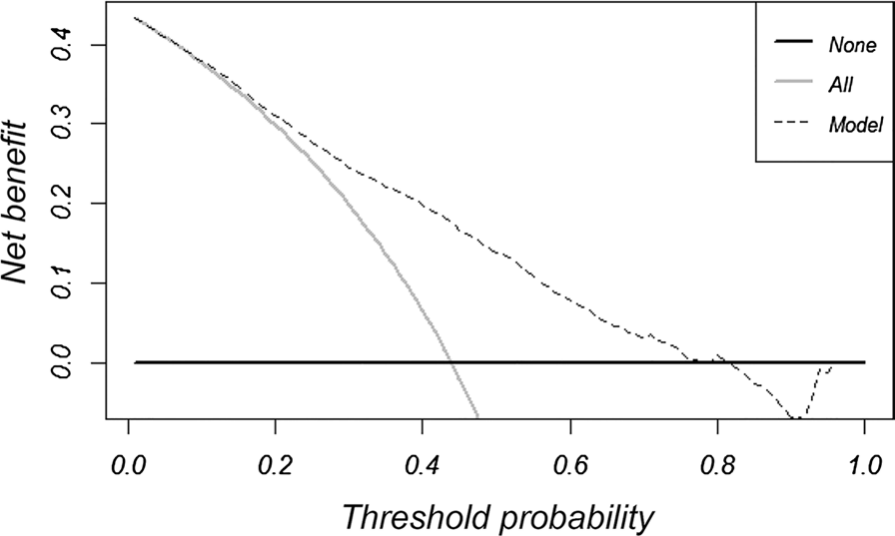
Decision curve analysis for classification of different risk population. The net benefit was calculated by subtracting the proportion of false positive from the proportion of true positive in all patients, weighting with the relative harm driven by false positive. Weighing the net benefit, threshold probability was classified for three-independent risk population, which was < 19.9%, 19.9–71.8% and > 71.8%, respectively

**Fig. 7:**
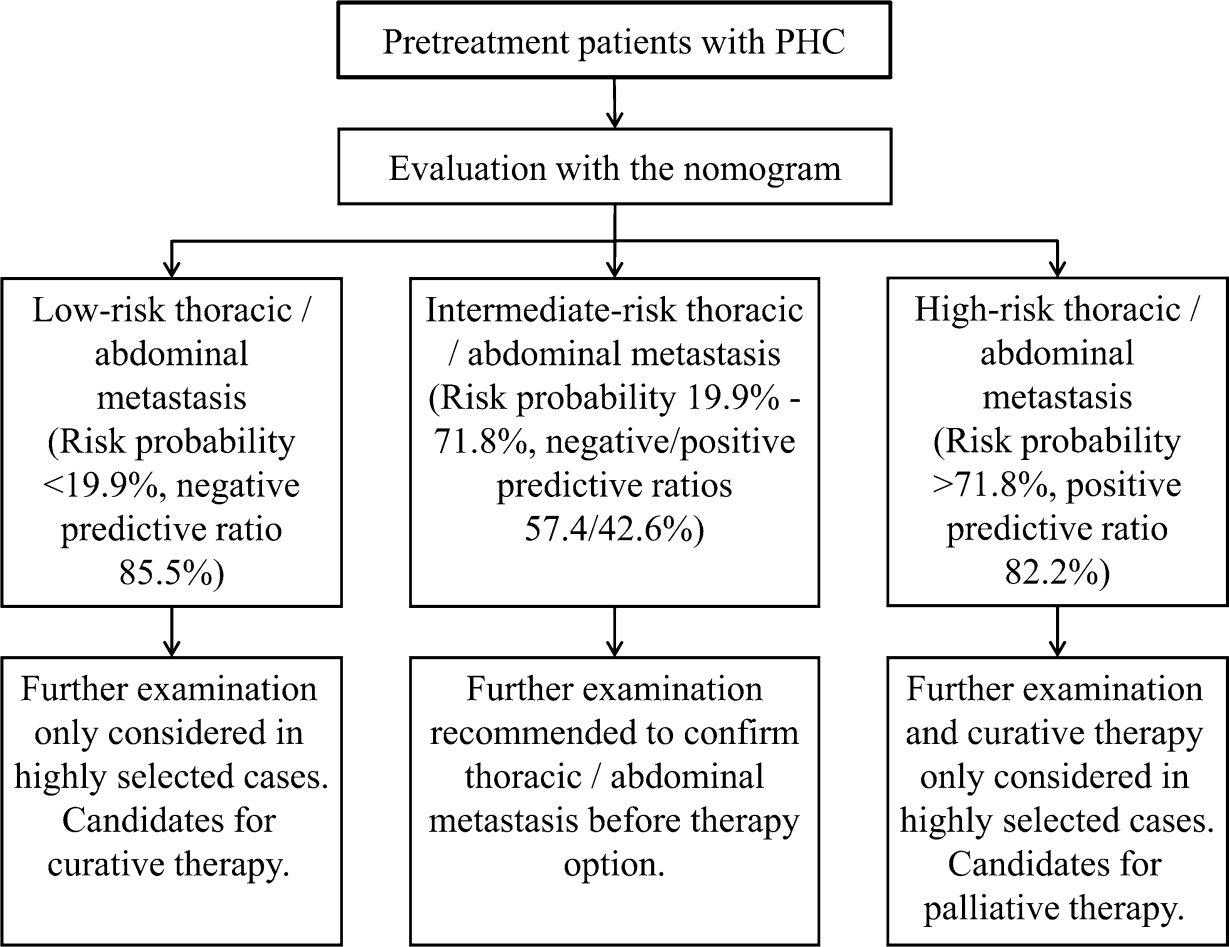
Treatment-flow chart of risk management for pretreatment patients with primary hepatic carcinoma (PHC)

DCA reported the risk threshold probability of the net benefit superior to the baseline ranged from 19.9% to 71.8% (Fig. 6). If the threshold probability is 30%, the net benefit is 0.247 superior to the treatment-all of 0.163 and treatment-none; then while the risk threshold probability is 15% (< 20.5%) and 85% (> 72.2%), the net benefit of 0.277 and − 1.388 are not superior to the reference strategies of treatment-all of 0.311 and treatment-none, respectively. Based on weighing the net benefit of differentiated threshold probability, the risk management may handily facilitate timely individualized clinical decisions-making for pretreatment different risk population (Fig. 7).

## Discussion

In this study, we developed and validated a nomogram model based on four clinical indices for individualized risk management of thoracoabdominal extrahepatic metastasis in pretreatment PHC. To our knowledge, this study is the first to develop and validate a predictive nomogram for thoracoabdominal extrahepatic metastasis in PHC based on large-scale datasets of multicenter 437 patients. The nomogram incorporated size, PVTT, infection and CA125, and demonstrated valuable prediction performance with AUROC of 0.830 (0.803 and 0.773 in internal and independent validations, respectively). Majority can be accurately predicted with accuracy of 77.8% (75.2% and 72.9% in internal and independent validations, respectively). Good calibration was observed in each set, suggesting that no departure can perfectly fit. Using the nomogram, the risk probability can be scored easily for thoracoabdominal extrahepatic metastasis in a patient with PHC. According to weighing the net benefit of individualized clinical decision-making, the differentiated risk management can be derived.

To develop a simple but efficient predictive model, we utilized the LASSO method to data dimension reduction and screen the optimized predictors. Four significantly independent predictors (size, PVTT, infection, and CA125) were selected from 55 collectable high-dimension clinical data for modeling. The LASSO is performed for both variable selection and regularization to enhance the accuracy and interpretability of the predictive model [10, 11]. This method surpasses the methods using the strength of univariate differences with outcome, and enables the most optimized predictors into modeling.

In the four predictors incorporated into the model, size and PVTT were proven to be independently related to extrahepatic metastasis of HCC [23–25]. One possible explanation is that tumor behavior, such as tumor size expansion, portal infiltration, or metastasis, is simply various manifestation of the same tumor stem cell with aggressive HCC biology [25]. The intrinsic association may involve angiogenesis. A retrospective study found that tumor survival, growth, and dissemination are dependent on angiogenesis along with microvessel density, which are significantly higher in patients with than those without distant metastases [26]. The principal route of recruitment of new blood vessels is as follows, tumor cells sustain growth, exit the primary sites, and enter the circulation [27, 28].

Recently, the relationship between infection and tumor metastases is a hotspot issue. Mantovani et al. [29] analyzed that “smoldering” inflammation in the tumor microenvironment has many tumor-promoting functions, such as supporting the proliferation of tumor cells, inducing angiogenesis, metastasis, and overturning adaptive immunoreactions. This may indicate that both infection and metastasis are of different aspects of immune imbalance in tumor. Matsumoto et al. [30] observed the decrease in the number and activity of natural killer (NK) cells in a murine liver metastasis model with induced abdominal infection; Kawarabayashi et al. [31] also found that decreased NK cells increased the susceptibility of bile duct-ligated mice to infection and tumor metastasis. Recently, some studies showed that complement promotes cancer metastasis through its contribution to epithelial-to-mesenchymal transition (EMT) [32, 33]. Mechanistically, tumor cells reduce their attachment to neighboring surroundings, increase motility, and acquire the invasive ability through EMT induced by the activation of complement receptors [34, 35].

Interestingly, as the “classic” biomarker for ovarian cancer, CA125 was a predictor of the nomogram. CA125 had been found as a tumor marker closely related to tumor metastasis, especially in gastrointestinal malignancies. Liu et al. [36] analyzed the serum levels of eight tumor markers, CA19-9, CEA, CA242, CA72-4, CA50, CA125, CA153, and AFP, in 1047 patients with pancreatic cancer and found that CA125 was the most strongly associated with the metastasis of pancreatic cancer and the expression of a metastasis-associated gene signature. The association of serum CA125 levels with metastasis had been observed in the liver metastasis of colorectal, [37] breast, [38] and lung cancer [39]. However, there have been no reports about the relationship between CA125 concentrations and PHC metastasis, although a few studies had observed significantly elevated serum CA125 concentrations in HCC [40] and ICC, [41] suggesting that other mechanisms may exist and more investigations should be conducted regarding the significance and mechanism of CA125 in extrahepatic metastasis of PHC.

The association of serum AFP concentration with PHC metastasis remains controversial. Some investigators reported that high AFP concentration was the adverse factors in extrahepatic metastasis of PHC [23–25]. However, Ogawa et al. [42] found no significant difference in AFP concentrations among the three groups patients with PHC (31 extrahepatic metastasis, 46 intrahepatic metastasis, and 14 no metastasis). Additionally, no significant correlation between circulating tumor cell number and serum AFP concentration [43]. Actually, none of these proved to be predictive, indicating that the mechanism of elevated serum AFP concentrations may differ somewhat from the distant metastasis of PHC. Serum AFP concentration, except for HCC, may be elevated during liver regeneration following hepatic resection and recovery from massive hepatic necrosis [44, 45]. Additionally, etiologies may also affect the serum AFP concentration. Adrian et al. [46] reported that the baseline AFP concentration was ≥ 20 ng/mL in 191 of 1145 patients (16.6%) with advanced chronic hepatitis C without HCC; simultaneously, the mean AFP values were also significantly higher in cirrhosis than in bridging fibrosis (22.5 vs. 11.4 ng/mL). Moreover, due to nearly 40% of patients with PHC are of AFP-negative (< 20 ng/mL), [47] the overall performance of AFP is easily disturbed and may be far from satisfactory as a metastatic marker.

We presented the nomogram model, which can visually score individual risk probability of PHC metastasis in the chest/abdomen according to four clinical predictors. For majority of patients with PHC, it is reasonable immediate tumor treatments after essential evaluation, thereby avoiding unnecessary surgical exploration and longer hospital stays in the high-risk population and excessive preoperative workups in the low-risk population. But for intermediate-risk population, further evaluation may drive more benefits, including comprehensive scanning of medical imaging and regular monitoring. The final value of the nomogram is to meet personalized clinical demands for different risk population. Therefore, DCA was employed in this study. This method provides insight into the clinical decision-making by weighing the net benefit at different risk threshold probability. Based on high negative predictive ratios (85.5%) in low-risk patients (risk threshold probability < 19.9%), further evaluation may not superior to immediate tumor-curative treatment as a result of these exhaustive workups cannot drive more benefit, while further evaluation should only be considered in highly selected cases. Similarly, for high-risk patients (risk threshold probability > 71.8%) with high positive predictive ratios (82.2%), gross thoracoabdominal metastasis portend a poor prognosis, and tumor-palliative treatment should be recommended for majorities while further evaluation and tumor-curative treatment may only be considered in highly selected cases. Patients who really benefit more from further evaluation should be intermediate-risk population between low-risk and high-risk patients (risk threshold probability from 19.9% to 71.8%). Due to the low positive/negative predictive ratios (42.6/57.4%), the net benefit during the risk threshold probability range superior to the baseline models of treatment-all and treatment-none. Both clinicians and patients could perform individualized risk management with this easy-to-use scoring system, which fits the current trend toward personalized medicine.

There are some limitations in our study. Firstly, the sample size in this study is not large enough, and because of incomplete data, some clinical parameters are not included such as hepatitis C virus (HCV). Secondly, the prediction performance of the nomogram is not enough excellent (especially in independent validation with AUROC < 0.8). As the first such study, there is no similar model for reference, and the proposed nomogram may be further optimized after incorporating more valuable variables and larger samples. Finally, as a retrospective study, we cannot avoid potential biases. Therefore, the reliability and stability of the nomogram remains to be further validated by prospective cases. In the next step, we will focus on conducting a prospective multi-center research for enrolling the large sample cases. In the prospective research, we will further evaluate the predictive value of variables that were valuable in reported studies but not into our model (including number of nodules, jaundice, hepatitis B surface antigen, HCV, Child–Pugh stage and so on) [23–25, 48], and strive to improve our model by optimizing predictors.

## Conclusion

In conclusion, this study systematically developed and validated a novel nomogram model for predicting thoracoabdominal extrahepatic metastasis in PHC. The proposed nomogram is a useful tool of pretreatment risk management for facilitating timely individualized clinical decision-making for different risk population.

## Additional file

**Additional file 1: Table S1.** Whole demographic and clinical data of all patients; **Table S2.** Characteristics of tumor and clinical conditions; **Table S3.** Concentrations and positive rates of four serum tumor markers between metastasis and non-metastasis groups.

## Abbreviations

PHC: primary hepatic carcinoma
HCC: hepatocellular carcinoma
ICC: intrahepatic cholangiocarcinoma
AFP: alpha-fetoprotein
LASSO: least absolute shrinkage and selection operator
DCA: decision curve analysis
CT: computed tomography
MRI: magnetic resonance imaging
SBP: spontaneous bacterial peritonitis
SIRS: systemic inflammatory response syndrome
CA: carbohydrate antigen
ROC: receiver operating characteristics
AUROC: area under the curve
PVTT: portal vein tumor thrombus
NK: natural killer
EMT: epithelial-to-mesenchymal transition
HCV: hepatitis C virus
