# Supplementary material for: Pretreatment risk management of a novel nomogram model for prediction of thoracoabdominal extrahepatic metastasis in primary hepatic carcinoma
Source: J Transl Med. 2019 Apr 8;17:117. doi: 10.1186/s12967-019-1861-z (PMC6454745; doi:10.1186/s12967-019-1861-z)
Supplement: Supplementary file 1 — Additional file 1: Table S1. Whole demographic and clinical data of all patients; Table S2. Characteristics of tumor and clinical conditions; Table S3. Concentrations and positive rates of four serum tumor markers between metastasis and non-metastasis groups. [file 12967_2019_1861_MOESM1_ESM.docx]

**Additional file 1**

**Additional file 1: Table S1** Whole demographic and clinical data of all patients

|  | Primary set | | | Independent-validation set | | |
| --- | --- | --- | --- | --- | --- | --- |
|  | Metastasis (n = 134) | Non-metastasis (n = 196) | *P* | Metastasis (n = 47) | Non-metastasis (n = 60) | *P* |
| Age (mean ± SD, years) | 56.8 ± 11.9 | 55.4 ± 11.8 | 0.302 | 60.6 ± 14.0 | 64.0 ± 12.3 | 0.182 |
| Gender [male/female, n (%)] | 97 (72.4) / 37 (27.6) | 165 (84.2) / 31 (15.8) | 0.009 | 37 (78.7) / 10 (21.3) | 47 (78.3) / 13 (21.7) | 0.961 |
| Etiological factors [(+), n (%)] | | | | | | |
| HBsAg | 81 (60.4) | 144 (73.5) | 0.013 | 38 (63.3) | 35 (74.5) | 0.220 |
| Alcohol | 38 (28.4) | 55 (28.1) | 0.953 | 15 (31.9) | 15 (25.0) | 0.429 |
| Hepatic function (mean ± SD) | | | | | | |
| ALT (U / L) | 57.9 ± 68.0 | 44.2 ± 56.0 | 0.055 | 64.5 ± 121.5 | 75.5 ± 89.6 | 0.589 |
| AST (U / L) | 97.7 ± 155.0 | 62.9 ± 66.7 | 0.015 | 152.9 ± 231.7 | 110.5 ± 95.3 | 0.243 |
| TBIL (μmol / L) | 31.9 ± 56.2 | 20.7 ± 28.1 | 0.034 | 52.3 ± 72.3 | 39.2 ± 43.9 | 0.248 |
| DBIL (μmol / L) | 18.9 ± 43.3 | 10.2 ± 23.4 | 0.035 | 31.5 ± 32.8 | 22.5 ± 32.1 | 0.156 |
| TP (g / L) | 67.5 ± 7.1 | 66.7 ± 7.0 | 0.311 | 64.4 ± 6.7 | 67.0 ± 9.0 | 0.102 |
| ALB (g / L) | 36.9 ± 6.4 | 38.9 ± 6.0 | 0.005 | 33.2 ± 6.4 | 35.1 ± 6.9 | 0.140 |
| GLB (g / L) | 30.3 ± 5.8 | 27.8 ± 5.4 | < 0.001 | 31.3 ± 7.3 | 31.7 ± 7.3 | 0.785 |
| AGR | 1.3 ± 0.3 | 1.5 ± 0.4 | < 0.001 | 1.2 ± 0.4 | 1.2 ± 0.4 | 0.852 |
| GGT (U / L) | 170.9 ± 227.1 | 110.4 ± 133.4 | 0.006 | 283.5 ± 365.2 | 210.2 ± 203.3 | 0.190 |
| AKP (U / L) | 205.4 ± 155.4 | 150.3 ± 117.6 | 0.001 | 198.6 ± 157.3 | 196.6 ± 139.3 | 0.944 |
| Renal function (mean ± SD) | | | | | | |
| BUN (mmol / L) | 5.5 ± 2.7 | 5.9 ± 3.4 | 0.362 | 6.5 ± 2.8 | 6.7 ± 4.7 | 0.736 |
| Cr (μmol / L) | 68.6 ± 21.0 | 76.4 ± 73.8 | 0.232 | 87.5 ± 41.2 | 92.7 ± 43.5 | 0.527 |
| Serum electrolyte (mean ± SD, mmol / L) | | | | | | |
| K^+^ | 4.2 ± 0.6 | 4.1 ± 0.5 | 0.126 | 4.3 ± 0.7 | 4.3 ± 0.6 | 0.965 |
| Na^+^ | 139.0 ± 4.1 | 140.3 ± 2.9 | 0.002 | 135.6 ± 6.7 | 138.6 ± 5.7 | 0.014 |
| Cl^-^ | 100.2 ± 4.8 | 102.1 ± 4.1 | < 0.001 | 97.0 ± 6.6 | 99.7 ± 5.0 | 0.018 |
| Ca^2+^ | 2.4 ± 0.2 | 2.3 ± 0.2 | 0.369 | 2.2 ± 0.2 | 2.2 ± 0.2 | 0.719 |
| Fasting blood glucose (mean ± SD, mmol / L) | 5.4 ± 1.6 | 5.3 ± 1.6 | 0.889 | 5.3 ± 1.6 | 5.9 ± 2.2 | 0.101 |
| Blood lipid (mean ± SD, mmol / L) | | | | | | |
| Cholesterol | 4.3 ± 1.4 | 4.0 ± 1.1 | 0.067 | 4.5 ± 2.5 | 4.4 ± 2.6 | 0.902 |
| Triglyceride | 1.2 ± 0.7 | 1.0 ± 0.7 | 0.107 | 1.3 ± 0.9 | 1.0 ± 0.6 | 0.821 |
| HDL | 1.0 ± 0.5 | 1.1 ± 0.4 | 0.070 | 1.0 ± 0.6 | 0.9 ± 0.4 | 0.455 |
| LDL | 2.6 ± 1.2 | 2.4 ± 0.9 | 0.048 | 3.1 ± 2.0 | 2.8 ± 1.2 | 0.427 |
| Coagulation function (mean ± SD) | | | | | | |
| PT (s) | 12.6 ± 2.2 | 12.4 ± 1.8 | 0.474 | 13.9 ± 4.0 | 13.1 ± 2.3 | 0.202 |
| PTA (%) | 83.6 ± 24.8 | 85.7 ± 20.9 | 0.410 | 70.2 ± 23.7 | 74.9 ± 22.7 | 0.301 |
| APTT (s) | 32.3 ± 8.1 | 32.7 ± 7.3 | 0.581 | 34.2 ± 8.2 | 35.9 ± 4.7 | 0.174 |
| INR | 1.1 ± 0.2 | 1.1 ± 0.2 | 0.514 | 1.2 ± 0.4 | 1.2 ± 0.2 | 0.294 |
| Blood cell analysis (mean ± SD) | | | | | | |
| WBC (× 10^9^ / L) | 7.0 ± 4.3 | 5.8 ± 3.5 | 0.004 | 7.3 ± 5.2 | 7.0 ± 2.8 | 0.694 |
| RBC (× 10^12^ / L) | 4.2 ± 0.8 | 4.2 ± 0.8 | 0.746 | 3.6 ± 0.8 | 3.9 ± 0.8 | 0.119 |
| Hb (g / L) | 126.6 ± 25.3 | 128.6 ± 25.2 | 0.475 | 106.5 ± 23.4 | 113.3 ± 20.5 | 0.110 |
| NEUT (× 10^9^ / L) | 5.0 ± 3.9 | 3.8 ± 3.3 | 0.002 | 5.4 ± 4.6 | 5.0 ± 2.7 | 0.496 |
| PLT (× 10^9^ / L) | 210.1 ± 130.7 | 167.0 ± 97.4 | 0.001 | 154.9 ± 102.8 | 172.7 ± 97.6 | 0.363 |
| Liver function status | | | | | | |
| C-P score (mean ± SD) | 6.6 ± 2.0 | 6.1 ± 1.7 | 0.011 | 7.7 ± 2.3 | 7.0 ± 2.5 | 0.160 |
| Grade A [n (%)] | 78 (58.2) | 138 (70.4) | 0.046 | 19 (40.4) | 338 (55.0) | 0.298 |
| Grade B [n (%)] | 38 (28.4) | 44 (22.4) |  | 15 (31.9) | 16 (26.7) |  |
| Grade C [n (%)] | 18 (13.4) | 14 (7.1) |  | 13 (27.7) | 11 (18.3) |  |
| ALBI score (mean ± SD) | -2.3 ± 0.7 | -2.5 ± 0.6 | 0.002 | -1.8 ± 0.7 | -2.0 ± 0.7 | 0.144 |
| Grade 1 [n (%)] | 61(45.5) | 108(55.1) | 0.115 | 8(17.0) | 15(25.0) | 0.229 |
| Grade 2 [n (%)] | 58(43.3) | 76(38.8) |  | 23(48.9) | 33(55.0) |  |
| Grade 3 [n (%)] | 15(11.2) | 12(6.1) |  | 16(34.0) | 12(20.0 |  |

**Note:** *P* value is derived from the univariate association analyses between metastasis group and non-metastasis group.

**Abbreviations:** SD: standard deviation; HBsAg: hepatitis B surface antigen; ALT: alanine aminotransaminase; AST: aspartate aminotransaminase; TBIL: total serum bilirubin; DBIL: direct serum bilirubin; TP: total serum protein; ALB: serum albumin; GLB: serum gamma-globins; AGR: albumin globulin ratio; GGT: glutamyl transpeptidase; ALP: alkaline phosphatase; BUN: blood urea nitrogen; Cr: creatinine; K^+^: Serum potassium; Na^+^: serum sodium; Cl^-^: serum chlorine; Ca^2+^: serum calcium; HDL: high density lipoprotein; LDL: low density lipoprotein; PT: prothrombin time; PTA: prothrombin activity; APTT: activated partial thromboplastin time; INR: international normalized ratio; WBC: white blood cell; RBC: red blood cell; Hb: hemoglobin; NEUT: neutrophils; PLT: platelet; C-P: Child-Pugh grade; ALBI: Albumin-Bilirubin score.

**Additional file 1: Table S2** C**haracteristics of tumor and clinical conditions**

|  | Primary set | | | Independent-validation set | | |
| --- | --- | --- | --- | --- | --- | --- |
|  | Metastasis (n = 134) | Non-metastasis (n = 196) | *P* | Metastasis (n = 47) | Non-metastasis (n = 60) | *P* |
| Primary tumor | | | | | | |
| Size (mean ± SD, cm) | 7.6 ± 3.9 | 5.5 ± 3.6 | < 0.001 | 7.7 ± 3.6 | 5.9 ± 4.0 | 0.018 |
| Multifocal [n (%)] | 73 (54.5) | 66 (33.7) | < 0.001 | 34 (72.3) | 28 (46.7) | 0.008 |
| PVTT [n (%)] | 86 (64.2) | 60 (30.6) | < 0.001 | 16 (34.0) | 8 (13.3) | 0.011 |
| Clinical condition [n (%)] | | | | | | |
| Ascites | 61 (45.5) | 65 (33.2) | 0.023 | 26 (55.3) | 18 (30.0) | 0.008 |
| Cirrhosis | 65 (48.5) | 98 (50.0) | 0.790 | 24 (51.1) | 35 (58.3) | 0.453 |
| Infection | 35 (26.1) | 10 (5.1) | < 0.001 | 23 (48.9) | 14 (23.3) | 0.006 |
| Gastrointestinal hemorrhage | 6 (4.5) | 11 (5.6) | 0.647 | 2 (4.3) | 5 (8.3) | 0.397 |
| Hypertension | 16 (11.9) | 18 (9.2) | 0.419 | 14 (29.8) | 13 (21.7) | 0.337 |
| Diabetes | 9 (6.7) | 21 (10.7) | 0.215 | 2 (4.3) | 12 (20.0) | 0.017 |
| Metastatic site [n (%)] | | | | | | |
| Lymph node | 78 (58.2) | - | - | 18 (38.3) | - | - |
| Lung | 14 (10.5) | - | - | 7 (14.9) | - | - |
| Gastrointestinal tract | 7 (5.2) | - | - | 1 (2.1) | - | - |
| Adrenal gland | 5 (3.7) | - | - | 5 (10.6) | - | - |
| Bone | 4 (3.0) | - | - | 3 (6.4) | - | - |
| Pleuroperitonea | 2 (1.5) | - | - | 2 (4.3) | - | - |
| Multiple sites | 24 (17.9) | - | - | 11 (23.4) | - | - |

**Notes**: Size: the maximum diameter of intrahepatic lesions.

**Abbreviations:** SD: standard deviation; PVTT: portal vein tumor thrombus.

**Additional file 1: Table S3** **Concentrations and positive rates of four serum tumor markers between metastasis and non-metastasis groups**

|  | Primary set | | | Independent-validation set | | |
| --- | --- | --- | --- | --- | --- | --- |
|  | Metastasis (n = 134) | Non-metastasis (n = 196) | *P* | Metastasis (n = 47) | Non-metastasis (n = 60) | *P* |
| AFP (ng / mL) | | | | | | |
| Concentrations (mean ± SD) | 476.6 ± 680.1 | 384.9 ± 513.3 | 0.187 | 511.4 ± 735.9 | 422.6 ± 651.2 | 0.510 |
| Positive rates [n (%)] | | | | | | |
| >= 20 | 70 (52.2) | 108 (55.1) | 0.608 | 32 (68.1) | 35 (58.3) | 0.301 |
| >= 200 | 57 (42.5) | 71 (36.2) | 0.248 | 26 (55.3) | 35 (58.3) | 0.755 |
| >= 400 | 84 (62.7) | 132 (67.3) | 0.382 | 32 (68.1) | 40 (66.7) | 0.877 |
| CEA (ng / mL) | | | | | | |
| Concentrations (mean ± SD) | 28.3 ± 127.0 | 4.0 ± 11.0 | 0.029 | 33.6 ± 78.4 | 7.5 ± 21.5 | 0.031 |
| Positive rates [> 4.6*, n (%)] | 47 (35.1) | 30 (15.3) | < 0.001 | 23 (48.9) | 41 (68.3) | 0.042 |
| CA19-9 (U / mL) | | | | | | |
| Concentrations (mean ± SD) | 200.4 ± 673.9 | 90.3 ± 286.2 | 0.076 | 246.4 ± 425.5 | 129.9 ± 310.3 | 0.119 |
| Positive rates [> 36.6*, n (%)] | 69 (51.5) | 52 (26.5) | < 0.001 | 28 (59.6) | 43 (71.7) | 0.189 |
| CA125 (U / mL) | | | | | | |
| Concentrations (mean ± SD) | 138.1 ± 249.9 | 85.5 ± 218.9 | 0.049 | 292.3 ± 349.5 | 170.3 ± 268.7 | 0.051 |
| Positive rates [> 13.9*, n (%)] | 111 (82.9) | 100 (51.0) | < 0.001 | 24 (51.1) | 43 (71.7) | 0.029 |

**Notes**: *: best cut-off value according to receiver operating characteristic (ROC) curve in primary study.

**Abbreviations:** SD: standard deviation; AFP: alpha-fetoprotein; CEA: carcino-embryonic antigen; CA: carbohydrate antigen.
